# Documenting the development, adoption and pre-ebola implementation of Liberia’s integrated disease surveillance and response (IDSR) strategy

**DOI:** 10.1186/s12889-023-17006-7

**Published:** 2023-10-25

**Authors:** Tolbert G. Nyenswah, Laura Skrip, Mardia Stone, Jessica L. Schue, David H. Peters, William R. Brieger

**Affiliations:** 1 Department of International Health, Johns Hopkins Bloomberg School of Public Health, Baltimore, MD USA; 2 School of Public Health, University of Liberia, Monrovia, Liberia; 3 Division of Global Psychiatry, Boston University School of Medicine, Boston Medical Center, Boston, USA; 4 Faculty of Health, York University, Toronto, Canada

**Keywords:** Ebola Virus Disease, Health Management Information System, Integrated Disease Surveillance and Response, Public Health Emergency, Liberia, West Africa, Health System strengthening

## Abstract

**Background:**

In the immediate aftermath of a 14-year civil conflict that disrupted the health system, Liberia adopted the internationally recommended integrated disease surveillance and response (IDSR) strategy in 2004. Despite this, Liberia was among the three West African countries ravaged by the worst Ebola epidemic in history from 2014 to 2016. This paper describes successes, failures, strengths, and weaknesses in the development, adoption, and implementation of IDSR following the civil war and up until the outbreak of Ebola, from 2004 to early 2014.

**Methods:**

We reviewed 112 official Government documents and peer-reviewed articles and conducted 29 in-depth interviews with key informants from December 2021 to March 2022 to gain perspectives on IDSR in the post-conflict and pre-Ebola era in Liberia. We assessed the core and supportive functions of IDSR, such as notification of priority diseases, confirmation, reporting, analysis, investigation, response, feedback, monitoring, staff training, supervision, communication, and financial resources. Data were triangulated and presented via emerging themes and in-depth accounts to describe the context of IDSR introduction and implementation, and the barriers surrounding it.

**Results:**

Despite the adoption of the IDSR framework, Liberia failed to secure the resources—human, logistical, and financial—to support effective implementation over the 10-year period. Documents and interview reports demonstrate numerous challenges prior to Ebola: the surveillance system lacked key components of IDSR including laboratory testing capacity, disease reporting, risk communication, community engagement, and staff supervision systems. Insufficient financial support and an abundance of vertical programs further impeded progress. In-depth accounts by donors and key governmental informants demonstrate that although the system had a role in detecting Ebola in Liberia, it could not respond effectively to control the disease.

**Conclusion:**

Our findings suggest that post-war, Liberia’s health system intended to prioritize epidemic preparedness and response with the adoption of IDSR. However, insufficient investment and systems development meant IDSR was not well implemented, leaving the country vulnerable to the devastating impact of the Ebola epidemic.

**Supplementary Information:**

The online version contains supplementary material available at 10.1186/s12889-023-17006-7.

## Introduction

Experiences from recent epidemics, including Ebola, Zika, Lassa fever, meningitis, and the COVID-19 pandemic, have highlighted the crucial role of public health surveillance systems in detecting emerging health threats, monitoring disease trends, and interrupting disease spread. The capacity of national health information systems (HIS) to collect reliable and comprehensive information is essential for effective operation of surveillance systems [1, 2]. A strong HIS is needed to ensure timely detection and decision-making around emerging health threats and is central to overall health systems strengthening.

The deficiency of public health surveillance data plus the need to improve disease control and prevention in Africa led the World Health Organization (WHO) Africa (AFRO) Region and the US Centers for Disease Control and Prevention (CDC) to propose an integrated disease surveillance (IDSR) framework in 1998 [3, 4]. The IDSR strategy was developed in response to a series of emerging and re-emerging diseases, which were responsible for the death of a significant number of people in the African Region [5–8]. While the COVID-19 pandemic has recently prompted considerable interest for national governments and international agencies to invest in disease surveillance and HIS [9], previous pandemic events offered lessons on surveillance that may not have been fully learned and applied. The Ebola outbreak in West Africa is one such event.

While lessons have been drawn from specific components of disease surveillance, [10] there has been no investigation into how the internationally recommended and donor-supported IDSR strategy may have lacked logistical, operational, and human resources capacities to prevent, detect, and control public health threats. In this paper, we document and describe how Liberia adapted, espoused, and implemented the IDSR framework in the post-civil war and pre-Ebola period from 2004 to 2014. We highlight which factors accelerated or hindered its development and explore why a decade after launching IDSR the health system was so severely disrupted by the Ebola epidemic.

## Methods

We conducted a case study of Liberia’s disease surveillance, epidemic preparedness, and response strategy for the period from its launch in 2004 till the onset of the Ebola epidemic in early 2014 (i.e., until January 2014, ahead of the March 2014 outbreak of EVD). Our qualitative approach considered two sources of data, (1) a document/desk review and (2) in-depth interviews with key informants. The two data sources were used to ensure comprehensive results and allow for triangulation of data as a standard method of case study research [11, 12].

### Brief description of Liberia’s surveillance system

IDSR implementation in Liberia is structured into tiers of health services delivery points and is the responsibility of all levels of the health system. From the lowest level at both the community and health facilities to the upper levels by the district and national actors. At the lowest level, there is community event-based (CEB) surveillance being implemented by trained community health assistants and Community Health Services Supervisor (CHSS), serving as the community disease surveillance officers. Liberia has 92 health districts unevenly distributed among the 15 counties, each having district surveillance officers (DSO). The DSO supervises disease surveillance activities in each district and reports to the County Surveillance Officers (CSO). The 15 CSOs are the third tier of the surveillance system and coordinate disease surveillance activities at the county level and report to the national surveillance system.

In Liberia, disease surveillance information is reported in a hierarchical order from the communities, through districts, and counties to the national health system. The regular flow of surveillance data is from each reporting sites (e.g., communities or health facilities) to immediate supervisors.

### Document /desk reviews

We carried out a document/desk review of official policy documents and guidelines (physical or electronic copies, depending on availability) and also reviewed both grey literature and peer-reviewed articles to understand the development, adoption, and implementation of IDSR. We obtained permission and signed data-sharing agreements with the Government of Liberia, Ministry of Health (MOH), and National Public Health Institute of Liberia (NPHIL) to use selected documents including conference or project reports, program logs, performance ratings, funding proposals, meeting minutes, newsletters, and quarterly reports. Three health-focused databases, PubMed, SCOPUS, and Medline were searched to identify grey literature and peer-reviewed articles (Appendix 1 diagram of five steps of systematic literature search). For electronic sources, a structured thesaurus and using text words/key search terms focused the research. Documents identified in the search were reviewed for inclusion based on the following pre-defined criteria. First, the document should be an existing government document from 2004–2014 and second, should contain key terms such as ‘IDSR’, ‘public health surveillance’, ‘public health security’, ‘outbreaks’, ‘disease surveillance,‘ and ‘infectious disease epidemics/pandemics’.

### In-depth interviews

The second source of evidence came from key informant interviews. We identified key informants as people who designed or were implementing IDSR in Liberia before the Ebola outbreak. We then conducted in-depth interviews with each selected key informant from December 2021 to March 2022 to gain perspectives on areas of IDSR effectiveness and factors that impeded implementation. An interview guide was developed (Appendix 2) but interviewers were encouraged to ask questions outside of the guide to explore IDSR-related experiences in-depth. At the start of each interview, participants were asked a series of demographic questions and the responses were summarized to characterize the study sample. Three persons conducted the interviews: the principal author, an epidemiologist (the co-PI), and an anthropologist. All three persons were trained in both qualitative and quantitative research techniques and field tested the instruments. Interviews were done in the English language, recorded, and later transcribed. Interview tools and approaches remained the same throughout the interview process to maintain consistency.

### Sampling strategy, participants enrollment and recruitment

We adapted a strategic, selective, and purposive sampling strategy [13–15]. Key informants were selected based on four categories as outlined in Table 1. The time category identified respondents who participated in the adoption or implementation phase of IDSR. The location category consisted of community, health center, or higher administrative levels and the phase category corresponded to what part of IDSR planning or implementation a respondent took part in. The status category identified the various roles the respondents played in the health system. Key informants were identified through registries, databases, directories, and listings of partner organizations (Appendix 3) sample size and frame.

**Table 1:** Key informant selection categories

Time Period	Location	Number of respondents	Phase	Status
2004 (IDSR adoption)	Community	5	Planning	Current Health Sector Coordinating Committee (HSCC) member
2005–2014 (IDSR implementation)	Health Facilities	680	Implementation	Previous HSCC member
2004–2014 (IDSR 1^st,^ 2nd editions training)	Districts	73	HIS training	
	Counties	15	IDSR capacity building	Multisectoral collaboration and partnership
	National	15	Supervision/ assessment	
			Data analyses	
			IDSR guideline development	Government staff representing non-health sector such as agriculture, livestock, and education
		10	Laboratory and training	locally registered non-governmental organizations (NGOs), and internationally registered NGOs
		5	Research	Donors

A key informant met the study’s inclusion criteria if they had an active role in the planning, implementation, and/or assessment of HIS/IDSR over the period covering 2004 through 2014. People were excluded if they had no experience in the surveillance and training activities of IDSR and did not implement activities related to IDSR.

### Researcher positionality/reflexiveness

Researchers bring their own biases and perceptions to the data gathering and interpretation processes, therefore, it is necessary to adopt reflexiveness and compassion to reduce the effects of these biases and the power dynamics between interviewers and interviewees [16]. In this study, the principal author played a key role in Liberia’s health sector which could influence how interviewees responded and how responses were interpreted. To address this in data gathering, two of the interviewers were not familiar with the study informants/participants, and the principal author carefully chose the respondents he would interview. We assessed participants’ authentic experiences on IDSR, took notes, and developed memos during the interviews processes.

The procedures, methods, attitudes, and orientations were adapted to consider reflexiveness, to avoid biases in what was said and heard, and to ensure the data were analyzed with a keen awareness of potential personal biases. Detailed records were maintained including journal entries of in-depth interviews where interviewers reflected on how and when personal experiences influenced research processes as a means of strengthening the confirmability of the data and maintaining transparency around the research methods [16].

### Data analysis

Qualitative research studies involve a continuous interplay between data collection and data analysis. Therefore, data analysis began following the first interview to identify emerging patterns and to facilitate subsequent data collection [17, 18]. The analysis process followed procedures suggested by Creswell (2009) and Esterberg (2002) [19, 20]. Data were examined during the open coding process using ATLAS.ti22 software (©2023 ATLAS.ti Scientific Software Development GmbH, Berlin, Germany) and codes were reviewed for emerging themes.

To ascertain a clear sense of this research, corroborate our findings, and address limitations, we triangulated data from the document review with representative quotes from key informants as the primary sources of evidence. Data from interviews and syntheses of documents were interwoven. When representing quotes from interviews, we assigned numbers to the categories of participants and cited each parenthetically (e.g., respondent Govt./MOH/MOA, Donor, NGO #001, 002, 003, 004, etc.).

From the literature, we anticipated reaching data saturation after 10–15 participants [21, 22]. Based on our sampling strategy, 29 participants were initially identified and approached for participation, and 29 were interviewed. All interviews were coded to understand the adoption and implementation of the IDSR strategy from 2004 to 2014 and to collect examples of usual or deviant observations [23]. However, no new topics, themes, or information were identified following analysis of approximately two-thirds of the interviews.

## Results

The results are presented in subsections that describe the context in which IDSR introduction and implementation occurred, highlight barriers to IDSR implementation in post-conflict Liberia, and give in-depth accounts of the surveillance system ahead of the EVD outbreak. Quotations were reproduced verbatim throughout the report in the respondents’ own words.

### Summary of documents reviewed and key informant interview participants

The number of government documents, grey literature, and peer-reviewed articles found during the desk review are summarized in Table 2. We selected documents from a pool of 166 official government documents and peer-reviewed articles: 112 of which met the inclusion criteria (i.e., published in the post-war, pre-Ebola period) and 76 did not. (Appendix 4 details the relevant documents identified from MOH, NPHIL and agencies).

**Table 2:** documents/desk reviews search results

	PubMed	Scopus	Medline	Official government documents	Total publications, across all databases/sources
Search results*	72	59	22	35	166
Search results: met criteria	43	35	13	21	112
Search results: did not meet criteria	29	24	9	14	76

* Based on the following search terms: ‘Liberia’s Ministry of Health’, ‘Ministry of Health and Social Welfare’, ’IDSR’, ‘public health surveillance’, ‘public health security’, ‘outbreaks’, ‘disease surveillance’, ‘infectious disease epidemics/pandemics”

Key informant interview participants included medical doctors, public health experts, field epidemiologists, community members, directors of programs or agencies, senior MOH officials (past and present), deans of schools of public health (past and present), and social scientists. Most key informants reported having 11–15 years of professional experience. The median age category was 40–44 years with a range of 30–65 years and females constituted 12 out of 29 persons. In terms of their location, 19 out of 29 persons were based in greater Monrovia. We had regional representation of key-informants from each of Liberia’s five health regions: northwestern, southcentral, southeastern A, southeastern B and north central regions.

### Demographics and background characteristics of study participants

Table 3 below shows the demographics of key informants Interviewed. Participants were those who participated in the introduction and implementation of IDSR. They were experienced in surveillance and related work and represented diverse backgrounds in the following disciplines: public health, medicine, health security, medical epidemiologist, health information system specialists, and public health disease surveillance officers. Participants were health professionals, program managers or administrators, and community leaders.

All 29 interviews were recorded using Zoom. Six participants were interviewed in-person and recorded. We spent approximately 90 min with each participant, accumulating approximately 2,700 min in interview time.

**Table 3:** Demographics of key informant interview participants

Variables	Frequency (n = 29)	Percentage %
**Sex**		
Male Female	17 12	58.6 41.4
Age in years (Mean age = 48 years ± 8.8)		
<39 40–49 50–59 60+	5 15 6 3	17.3 51.7 20.7 10.3
**Classification**		
National International	24 5	82.8 17.2
**Cadre of work**		
Medical doctor Field Epidemiologist Community member Public Health Managers/Experts	6 5 4 14	20.7 17.2 13.8 48.3

### Contextual factors surrounding IDSR introduction and implementation

This section describes the factors that impacted the development, adoption, and implementation of IDSR. We identified five themes from the period 2004–2014 based on our analyses of data from key informant interviews and the desk review. These included (1) the history of surveillance in Liberia, (2) the health information system (HIS) within which surveillance took place, (3) multiple concurrent health security and disease threats, (4) community engagement and risk communication, and (5) human resources and capacity building needed for surveillance. In addition to the logistical and organizational needs for structured surveillance, the five themes reflected the overarching epidemiological, sociocultural, political, and economic context.

### Theme 1: history of surveillance in Liberia

In 1998, the major weakness in national health surveillance and response systems in many African countries, included Liberia had been widely recognized [24]. In response, the WHO African region (WHO AFRO) and US CDC proposed the IDSR strategy to strengthen public health surveillance and response systems, which ultimately led to the introduction of IDSR in Liberia in 2004. All key informants and documents we reviewed confirmed that IDSR was adopted in Liberia in 2004 and that some policy guidelines were developed. Most MOH officials and representatives of international organizations corroborated that in each of the 15 counties where IDSR was introduced, there was only one staff member assigned to implementation. Other activities, such as training, were ongoing. Logistics (motorcycles etc.) were procured using acute flaccid paralysis surveillance funding.

A key informant from a donor agency explained that,

*“…Liberia was the third country in Africa, at that time, to adapt the IDSR strategy and guidelines in 2004. Although the adapted strategy was a beautiful document, the systems and structures needed to support the rollout of the strategy was limited. This was another conversation, apparently, several issues came about*: (1) *resources were not there in the Ministry of Health*, (2) *the human resource capacity was still limited*, (3) *in the counties, things were disjointed, because the county health teams were not quite robust at that time. So, the entire introductory phase had its own drawbacks.” Donor#009*.

Government employees described implementation strengths and weaknesses during this early period.

*“… And to be honest, we appreciated what was happening from the field. The surveillance officers were trying as much as they could, but I think there were more challenges to really look at the functionality of IDSR.” Govt./MOH#002*.

*“…so, first thing I would say is, in 2013, prior to that time, the strengths in implementing IDSR was that the health care system had a dedicated unit in the MOH. That unit was connected and linked to the WHO as the closest partner. There was a system in place. As faulty as it was, facilities were reporting to central level. Ministry of Health invested in training dedicated staff for those roles and responsibilities. Liberia had just emerged from a civil war, so all these issues were not on the government’s radar.” Govt./MOH #003*.

### Theme 2: health information system (HIS)

An expert in the field of health information systems described the DHIS2 system and how this HMIS system was a gamechanger,

*“…it is considered as the world’s largest information system, developed through global collaboration led by the University of Oslo, in Norway.” Govt./MOH #003*.

*“this system is efficient and make reporting simpler as compared to the paper-based system we had before.” Govt./MOH #003*.

*“…in Liberia there are subsystems that feed into the HMIS, integrated disease surveillance response system; vital registration (death and birth); health information system (HIS-using district health information system version two -DHIS-2); logistic management information system (LMIS); and human resource information system (HRIS).” Highlighting, “a functional surveillance system will generate data or information needed for planning, implementation of public health interventions and, to evaluate public health practices.” Donor #026*.

### Theme 3. multiple health security threats

The Joint National Action Plan for Health Security (NAPHS) emphasized the concurring and persistent threats that Liberia has experienced. When Liberia experienced the unprecedented Ebola virus disease outbreak there were already several concurrent disease threats including outbreaks of Lassa fever, monkeypox (now Clades I and II), rabies, and meningitis.

*“It was the matter of ‘when’ not, ‘if’ the fragile post-conflict health system would be caught off-guard and overwhelmed by the Ebola epidemic.” Donor #009*.

### Theme 4: community engagement and risk communication

Prior to the Ebola epidemic community engagement, health promotion and risk communication activities were scarce and centered around polio eradication initiatives. This made risk communication extremely challenging during the initial stages of the Ebola outbreak. Wildly spreading rumors and misinformation about the virus also adversely affected response efforts. Some communities were reluctant to report sick loved ones and relatives for fear of being quarantined or taken to the Ebola treatment unit (ETUs). More illnesses and deaths occurred at homes and secret burials were frequently practiced to avoid the government’s cremation policy. Hence, interrupting the transmission of the virus became complex.

It became imperative to introduce risk communication as a way of providing real-time information and easily understood messages to the public about Ebola and encouraging participation in response efforts. In October 2014, Liberia’s National Health Promotion Division and the response thematic group initiated Ebola related community engagement and risk communication activities. Risk communication considered multiple communication channels: individual interpersonal, community health workers, social mobilization, radio messaging, focus group discussion, and mass media. Messaging prompted people to observe preventive measures including, no shaking hands, washing hands with soap and water, not touching sick ones suspected of having the disease, allowing safe burials, identifying sick people within the community, and calling the EVD hotline to pick up sick people from the community.

Despite progress, the health promotion efforts continued to encounter misconceptions, doubts, and community resistance. A document from the US CDC — “Gateway to Health Communication” — emphasized how understanding cultural nuances and traditional practices of the African context is critical for surveillance and response. Native laws and customs are sacred and often perceived as rites of passage throughout an individual’s life in traditional society. Appropriate community engagement and risk communication are crucial in any outbreak response. Religious diversity in Liberia was one cultural nuance that had to be accounted for during disease response.

### Theme 5: human resources for health and capacity building

In this study we wanted to understand the existing capacities and capacity building activities around the financial, supervision, and logistical tasks in place in the Ministry of Health during the pre-Ebola period. This included the ability to support the hardware and software components of IDSR. We were also interested in the stakeholders’ involvement in providing these services as well. We describe the stakeholders, policies, and barriers that impacted human resource capacity for IDSR from 2004 to 2014 pre-Ebola.

*“…so, because the Government of Liberia, the Ministry of Health did not have the capacity, and limited policies, if they existed, were not implemented. The concentration at the time was to provide basic health services. Partners, including WHO, USAID, Merlin- were integral part.” NGO#027*.

Likewise, the health sector did not have the resources to support its workforce, which was growing but without formalization, such as inclusion of personnel on payroll, and with dependence on external support.

*A respondent said: “Issues of maintaining health workers salaries, caused three strike actions of health care workers, even before the Ebola crisis. This further exacerbated the Ebola situation on the health system.” Respondent continues “…so, one of the big mistakes we made, we kept hiring people, but those people did not become civil servants, meaning, they were not put on Government’s payrolls over time. NGOs were paying government workers salaries. This kind of system created problems of fragmentation and divided health system. Loyalty of workers was paid to whoever was paying their salaries, fragmenting, and over burdening the reporting system. Vertical programs staff were sending their reports to either GAVI, Fixed Access Reimbursable Agreement (FARA), a USAID financed health project, Global Fund, PMI, UNICEF or the Pool Fund.“ NGO#027*.

Moreover, respondents opined the general views that human resources after the war were depleted by migration, both internal and external. For instance, people left the public sector to work for NGOs. Starting from 2006, the MOH developed an incentive scheme to get its workforce back. However, that was still plagued by challenges due to inconsistent resource availability for regularly ensuring incentives.

### In-depth accounts of surveillance system

We recount two real-life events to demonstrate the status of the surveillance system at the start of the Ebola outbreak. In-depth accounts from key informants are provided. The informants for these accounts include one representing a donor organization (#007) and one from the MOH (#005); the others are surveillance officers representing the Ministry of Health’s five health regions, Govt./MOH (#016), (#017), (#018). The first respondent played a critical role during the introduction of IDSR and the Ebola epidemic, which makes his perspective noteworthy. We also describe the experiences of six regional surveillance officers, who were involved in the introductory stage of IDSR. They also participated in the Ebola response, and the IDSR revitalization, during and post Ebola recovery.

The first account comes from respondent donor #007:

*“…now, we are getting close to Ebola late December 2013, the reason, I could give credit to the health workers at that time is because, the rumors of Ebola at that time, we all had the conviction that Ebola was in Central Africa and East Africa at that time. There were no thoughts of Ebola being in the West Africa subregion. Although, after the Ebola crisis we saw literature that as early as the 70s there were cases in West Africa, even in Liberia and in Ivory Coast. …during the early stages of the Ebola crisis, I was performing two roles [at the donor organization]: 1) one being an epidemiologist, I actually became an officer now for disease prevention and control and* 2) *also an officer responsible for health security and emergencies. So, I had two portfolios at one time in 2013. I was acting for my boss at the time, who was on leave. That’s when I got the information regarding suspected cases from WHO Regional Office for Africa in Congo, Brazzaville, and I was informed it was Lassa fever, from Guinea. Knowing that Liberia was a Lassa fever belt, WHO wanted support from Liberia in terms of medicines, ribavirin. At the time, … Liberia had enough medication, so [the CMO] was happy to help Guinea by providing the drugs. But after two days, down the road, there was no other information from the WHO headquarters regarding the news I was given regarding the suspected Lassa fever in Guinea. I remember very well it was on a Friday. And I thought since Fridays are half days for the UN but can also be days that emergency can happen…so, then I volunteered to ask my regional office what was happening, because Friday being half day, there was no one telling me anything and later I would be able to get people to help me. Only to hear WHO colleagues in the region were talking about something else, maybe viral hemorrhagic fever, other than Lassa fever. So, really, I was a bit moved, so I called the Chief Medical Officer for an emergency meeting, and we all agreed that we send a team from our side in Liberia to the Guinea border. So, I had somebody from WHO, someone from the Ministry of Health, that joined with the Lofa county health team, to go and understand what was happening, though there was scant information, but really, from the case definitions in the IDSR guidelines, I could also commend them…this is what brings me to the issue of case investigation. The team was able to go and do record reviews and found out that really the cases that probably crossed from Guinea into the hospital in Foya, Lofa County, had similar symptoms of what was being reported in Guinea.“*

The informant’s account is a narrative of what appeared to be the origin of the EVD outbreak in Liberia. He continued:

*“…we followed up with patients, we noticed some had died, some crossed back into Guinea, then some of the contacts were around the city. We took the specimens of those contacts— they were positive for Ebola. Those samples were the first cases that were reported in Liberia…the beginning of the end of Ebola in Liberia in 2014.“*

The informant described the situation as disastrous. When asked why, the informant grieved:

*“…you know, when a system crumbles, you are really going through a crisis. So, from fewer cases there were already several countless graves of people who had died at that time. The villagers, community people documented the cadavers, odor all over the place, they kept a ledger and documented and kept records of people who died. The villagers were the real ‘shoe leather epidemiologists’. Whether people died in their own communities or in their homes, that community leaders-chiefs and Iman showed us the burial sites.” Donor #007*.

We managed to independently interview the Government/MOH representative (#005) who was part of the team that traveled to the Liberia-Guinea border per the donor participant’s account. He corroborated the account with more insight into the surveillance context of the visit to Lofa that uncovered Ebola in Liberia:

*“…no doubts, I can emphatically say the surveillance system was weak prior to the Ebola crisis. Imagine we had just introduced district surveillance officers; they had no logistics to work with. The Ministry, through my office, talked to WHO to provide incentives for surveillance officers, no knowledge base, we had zero ideas about Ebola in West Africa. We only read few articles and materials from elsewhere, for example, our messages were saying, Ebola is real, no cure, the case fatality rate is between 25–90%. Ebola was not a reportable disease. Guinea did not know they were dealing with Ebola. Guinea wrote Liberia through WHO, thinking they were dealing with Lassa fever. As the person responsible for disease prevention, the CMO sent me the letter, from Guinea, requesting for Lassa fever, Ribavirin drug. At the national level, there was no degree of public health leadership. My small team and I, few staff, no vehicle, we didn’t have essential supplies, no drugs, no PPEs, no ambulance. The old ambulance I took to Lofa, the epicenter of the Ebola, broke down several times before we got to Lofa, near the Guinean border…it was too pathetic! We did not have vehicles to bury the corpses we met on arrival in Foya, Lofa. No logistics. No diagnostic capacity for Ebola, we took the samples to Guinea, where MSF had a camp. In reality, Liberia did not have the capacity for surveillance emergency supplies. Infection prevention and control knowledge was seriously lacking. Zero health facilities management, no isolation to keep the infected patients, everybody was admitted everywhere on the patients’ walls. Really, logistics was a mess.” Govt./MOH #005.*

Regional surveillance officers representing the five health regions of the country (Northwestern, South Central, Southeastern A, Southeastern B and North Central) further added in-depth understanding on the environment for implementing IDSR in the context of policy, political, economic, and budgetary constraints in post-conflict Liberia. One respondent said:

*“…we did not have tools to work with, such as training materials…only few motorbikes with no regular spare parts provided by WHO… we walked long distances to collect surveillance data and specimens, only AFP surveillance was functioning…. we were not collecting data for the other diseases. When you transport AFP surveillance to Monrovia, WHO only gave you US$90 for fuel for the motorbike, no incentive at all*^*____*^*another aspect is that our salary was not regular; we were just working.” Respondent Govt./MOH#016 southeastern region.*

One officer from Southeastern A, with specific knowledge of the system indicated:

*“…people were not trained in how to identify and report on priority diseases… further we were missing out on cases because we did not have the capacity to respond, at the county and district levels. We could not do formal graphing, data analysis, [and we had] limited knowledge of the line listing of diseases…contact tracing and other outbreak response strategies just came about during the Ebola epidemic. To further complicate things, there was very limited logistical support such as: vehicle, internet system at the time, and motorbikes were broken down… for example, if you had a suspected case, to move the case to the hospital, you did not have a motorbike or ambulance system.” Respondent Govt./MOH#017 northern region.*

Another ministry informant mentioned that:

*“…just imagine, I was supervising 37 health facilities, no bikes to go to the facility and collect specimens to send them to the laboratory in Monrovia. I can tell you for sure, I will only get to the facilities when [Expanded Programme on Immunization] (EPI) folks are going for supervision, sometimes once a month. Additionally, the communities were not involved in disease surveillance at the time, now, during and after Ebola, community engagement was now key. We then introduced the general community health volunteer program (gCHV) in every community…it now includes, community surveillance system, though it was not working before the Ebola crisis, so it was really challenging.” Respondent Govt./MOH#018 northcentral region.*

### Barriers to IDSR implementation in post-conflict Liberia

Upon synthesizing findings from the desk review and key informant interviews, we identified nine key barriers that inhibited the implementation of IDSR from 2004 to 2014 which are summarized with their descriptions in Table 4. Illustrative examples aid in the understanding of what likely hampered the IDSR processes and implementation leading up to the Ebola outbreak.

**Table 4:** Key barriers of IDSR (2004–2014)

Issues	Brief Description
1. Disabling political, economic, and social environment	• 14 years (1989–2003) of large-scale armed conflict in Liberia caused massive destruction to the country’s infrastructure, systems, and social cohesion. The economic, social, and political environments were weakened. • The loss of human, social, and economic resources challenged the country after the conflict ended. • Insufficient economic activities impeded access to financial resources and thus affected re-establishment of the health and disease surveillance systems.
2. Unequipped human resources	• County surveillance officers were high school graduates who had carried out surveillance for acute flaccid paralysis (AFP). • Lack of salaries for surveillance workers. • 35% of the health facilities had only one county surveillance officer to collect, report, collate, and analyze surveillance data and information. • Lack of a field epidemiology training program and data management skills.
3. Donor-dependent financial support	• The entire surveillance system was donor dependent and driven. • Inadequacy of budgetary allocation in the MOH budget for surveillance activities. • Transportation reimbursement and daily sustenance allowance for surveillance officers was only provided by WHO’s country office. WHO also provided for surveillance workers.
4. De-prioritization of training and supervision	• Inadequate budgetary support from the national government for training, field investigation, and supervision of surveillance activities.
5. Vertical program reporting, lack of integrated surveillance system	• Only vaccine preventable diseases such as yellow fever, measles, polio, etc., were responded to because these were the only diseases reported to WHO through the expanded program on immunization for which DSA was provided to the surveillance officers.
6. Inadequate laboratory capacity	• Lack of testing capacity for priority notifiable diseases including yellow fever, Lassa fever, Ebola, and others. All AFP and Lassa fever samples were sent out of the country for testing. • No national reference laboratory
7. Weak surveillance structures in practice	• Lack of well-established surveillance structures from communities, districts, and counties to the national. • Symbolically there were structures, but they were not capacitated. • Non-existent incident management system teams. • No capacity for rapid response teams. • Non-existent emergency operations centers at the communities, districts, counties, and national levels.
8. Lacking active surveillance and reporting mechanisms	• Active surveillance was nonexistent. The surveillance system was reactive rather than being a proactive surveillance system to detect diseases of epidemic potential. • Reports were submitted through desk phones; surveillance officers made reports through a very high frequency (VHF) radio. Each county had a desk officer that collected the information and reported it to Monrovia. • No computer system, no data clerks.
9. Lack of logistics and equipment	• Insufficient vehicles and motorcycles. There was only 1 motorbike per county. • Lack of communication equipment. • No GSM network at the time. • No computers. • Surveillance system was handicapped for lack of logistical support. 15 gallons of gasoline were provided by WHO only when reports were delivered.

## Discussion

We found that the integrated disease surveillance approach was hindered by the presence of multiple vertical donor and humanitarian programs, such as HIV/AIDS, Tuberculosis (TB), Malaria Control Program, expanded program on immunization (EPI), and programs on neglected tropical diseases (NTDs). These vertical programs were funded by several donors including the Global Fund for AIDS, TB and Malaria, USAID, UNICEF, WHO, etc. They supported the delivery of basic health services, though not within an integrated framework. Consequently, surveillance and response activities to prevent, detect, and respond to public health threats was not a part of the basic and essential packages of health services, leaving the population susceptible to infectious disease threats, as was clear with the unchecked spread of Ebola across the country. Of significant concern was that human resources were unequipped to handle surveillance of a disease outbreak. Front-line staff consisted of county surveillance officers who were high school graduates and who had carried out surveillance for acute flaccid paralysis (AFP) as part of the polio program. There was lack of salaries for surveillance workers. 35% of the health facilities had only one county surveillance officer to collect, report, collate, and analyze surveillance data and information. In particular these staff lacked field epidemiology training and data management skills.

From 2004 to 2014, the decade preceding the Ebola Epidemic, Liberia’s health system extensively implemented vertical disease surveillance and response strategies for priority infectious diseases. Several drawbacks with Liberia’s health system included: the high cost of maintaining the various parallel systems; the inability of the several vertical disease surveillance strategies to adequately fulfil the functions of surveillance and response; heavily centralized systems; an inability to detect disease outbreaks in a timely manner; duplication of work due to lack of coordination between vertical programs; overburdened health personnel responsible for disease surveillance and so on. Additionally, well-documented challenges to the health system included: strengthening laboratory networks; routine monitoring and providing regular feedback and supervision on IDSR activities; and extending the strategy to county health teams and health facility-levels.

We demonstrate that in the absence of a strong health system in LMICs, diseases will thrive, causing unprecedented havoc on the human population, disrupting international trade, and impacting global health security. COVID-19 has highlighted this. On one hand, the pandemic has engulfed health systems as a whole, especially in low-income countries where they are most fragile. On the other hand, improved health systems can detect outbreaks before they become epidemics or pandemic. Donors must tailor their support to countries’ action plans for pandemic preparedness and response. They should enable countries to detect threats within their own borders, implement effective control measures, and forewarn other countries of the potential risks.

To fully integrate with national health systems, IDSR should be aligned with a country’s broader health information systems. This will require explicit efforts to ensure that the units responsible for IDSR are integrated within the units responsible for health management information systems.

### Fragility, conflict and IDSR

It is evident that the prolonged civil conflict, humanitarian crises, and public health emergencies caused disruption of health and other social services and therefore affected IDSR implementation in Liberia. Based on the experiences from other settings in the African Region, the second and third editions of the IDSR technical guidelines were revised to include several key components and lessons learned from implementing IDSR in humanitarian crises [24].

In 2004, the WHO Africa Region (AFRO) and US CDC recommended and introduced IDSR in Liberia; however, implementation of the system was handicapped. Despite the IDSR guidelines, the system did not incorporate all priority diseases. The surveillance system was centered around vaccine preventable diseases, which was not comprehensive nor well integrated. We uncovered that the funds provided for polio, a vaccine preventable disease, were used to support surveillance officers in general and not exclusively those directly involved in the polio surveillance network.

Respondents described the surveillance system as being inefficient, inadequate, and not permeating the health system at all levels. Nearly all mentioned that a functional IDSR strategy does not end at the facility level but reaches out into the community. Prior to the Ebola epidemic, the surveillance system did not include the community component, making it harder for surveillance workers to detect rumors before they become full scale outbreaks.

In other settings that have been marred by conflict, introduction of a large-scale, high-resource system warrants close attention. Its introduction needs to be scaled with adequate support at each step and political will to promote government ownership. Achieving systematic implementation of IDSR has been shown to take years, particularly in post-conflict settings, and revitalization is frequently undertaken to reflect evolving country needs [25–27]. Evaluations of IDSR suggest progress [24], yet indicators are often on a macro scale (e.g., whether or not a country is training at subnational levels) rather than indicators on which the system’s effectiveness and sustainability may be predicated (e.g., human and logistical resources at subnational level to routinely conduct training and assessments in place to evaluate quality of training) [26]. Strengths and weaknesses described here for Liberia and elsewhere for other SSA countries suggest recurring issues that both emphasize the need for IDSR and the challenges to implement it, leading to vulnerability.

### Strengths and limitations

The strengths of the research outpace the limitations. The study provides understanding of the evolution of IDSR strategy and its core surveillance functions including case detection, confirmation, reporting, analysis, investigation, response, feedback, and monitoring. The mixed method study design is a major strength, the study used qualitative information to guide the interpretation of findings and establish a range of views related to IDSR implementation. The case study elicited the perspectives and opinions of key stakeholders in the field of disease surveillance, preparedness, and response as sources of evidence that informed our findings and conclusions.

A major limitation of this study is that the principal author could not travel in person to the study setting. We intended to conduct in-person focus group interviews within Liberia and visit a few health facilities that collect surveillance data to supplement the key informants’ interviews. This was not possible due to Johns Hopkins University’s COVID-19 essential travel restrictions which prevented faculty, students, and staff from travelling during the height of the pandemic. Additionally, the US CDC raised Liberia’s travel alert to level 4, the highest risk level they assign, indicating that the country was highly unsafe for inbound travelers. We overcame these restrictions by deploying two methods. First, we asked highly skilled colleagues who were already stationed on the ground in Liberia to conduct some interviews. Second, the principal author of this study used replaceable electronic means, such as virtual interviews via Zoom. Another limitation was difficulties in locating interviewees. Some of the people we intended to interview were expatriates that left Liberia before study began. Additionally, the principal author’s reflexiveness and positioning still present a potential limitation. All efforts were made to minimize any bias introduced and are unlikely to affect the data collected, data analysis, results, or conclusions of this study.

## Conclusion

Liberia emerged from a period of conflict to rebuild its health system focused on service delivery, epidemic preparedness, and response. Our findings suggest that it was only a matter of time until Liberia’s health system would have been disrupted by a large-scale event such as the 2014–2016 Ebola epidemic. We document and describe Liberia’s unsystematic, weak, and unevaluated implementation of the IDSR strategy prior to the 2014–2016 EVD epidemic, which had devastating consequences with nearly 11,000 people infected and 4,810 people dead [28]. It highlights fragility, with gaps in areas such as community engagement and risk communication, HR capacity building, health infrastructure, coordination, and implementation.

Our findings demonstrate that the health information system, vis-à-vis the IDSR strategy as established, was vulnerable to the disastrous effects of the Ebola epidemic and any other novel high consequent pathogen of unknown origin. The Ebola epidemic prompted an opportunity for the revitalization of IDSR. A major focus of this revitalization should be integration, which may range from interconnectivity that requires a simple transfer of files with basic applications, to complex convergent integration, which involves merging technology with processes, knowledge, and human performance.

### Electronic supplementary material

Below is the link to the electronic supplementary material.

Supplementary Material 1

## Data Availability

The datasets used and/or analyzed during the current study are available from the corresponding author on reasonable request.
