# Supplementary material for: Documenting the development, adoption and pre-ebola implementation of Liberia’s integrated disease surveillance and response (IDSR) strategy
Source: BMC Public Health. 2023 Oct 25;23:2093. doi: 10.1186/s12889-023-17006-7 (PMC10601278; doi:10.1186/s12889-023-17006-7)
Supplement: Supplementary file 1 — Supplementary Material 1 [file 12889_2023_17006_MOESM1_ESM.docx]

##### Appendix 1 Diagram of five steps of systematic literature search

Adopted from: Saleh AA, Ratajeski MA, Bertolet M. Grey Literature Searching for Health Sciences Systematic Reviews: 2014;9(3):28-50. doi: 10.18438/b8dw3k. PMID: 25914722; PMCID: PMC4405801.

##### Appendix 2 depicting interview guide

| Introduction  Hello, my name is Tolbert Nyenswah DrPH student from Johns Hopkins Bloomberg School of Public Health. I appreicate the time you are taking to speak with me. I am interested in talking to you about your role and perceptions about the FETP in Liberia. This is in fulfilment of a case study that I am conducting on the effectiveness of the FETP in Liberia and how it supports the IDSR system.  A case study report will be produced using the data from the interview, document review as well as testimonials surveillance officers who benefitted from FETP training. |
| --- |
| Key Informant Interview questions  *CDC/WHO/AFENET and other parners*   - When did the CDC become involved in the FETP in Liberia? - Do you think it has met its intended purpose? - Can you briefly describe your relationship and roles with this program? - What, if any, amount of funding does your organizaiton provide to the FETP, either direct or indirect? - From your perspective as a partner to the Liberian Government, what challenges have you experienced in working on the FETP? - With the current structure of the program, do you think it is sustainable? - What, if any, are key lessons learned from your perspective? |
| Documents to be reviewed:   - NPHIL: National Strategic Planning document for - MOH:Investment Plan for Building a Resilient Health Care System. - Summary of progress reports by NPHIL and AFENET field office in Liberia, - AFENET web-page and annual reports |
| Testimonials from Surveillance officers, (FETP graduates) and Government Leadership (NPHIL, MOH, Surveilllance Officers)   - Since you became director of the program, surveillance officer, what has been your involvement with the FETP?   Can you describe your leadership roles, relationship and responsibility with the FETP   - What aspect of the program, are you engaged in, specifically… (training, data collection, analysis, reporting, identification or laboratory work)? - Has Government provided funding for the FETP? - How sustainable is the program, from you perspective? - Have you received training, if so, for how long? - What do you think is the future of the FETP? |

Sources: Adapted from the Macro International, Inc, interview guides in collaboration with

CDC Healthy Aging and PRC program. Appendix B1=B3^[[1]](#endnote-1)^

##### Appendix 3 Sample size and frame

| **Category** | **Sample Frame** | **Criteria** | **Initial selection*** |
| --- | --- | --- | --- |
| I. Government representing Health Sector   1. Multisectoral collaboration and partnership 2. FETP Program in the MOH/NPHIL | A registry of 34 present and past Health Sector Coordinating Committee (HSCC) members.  Database of FETP graduate about (n=248) FETP trainees representing Field Epidemiology Network. | - **Inclusion:** people who were part of the development, deployment of IDSR, Ebola response and rebuilding of a resilient Health System. - **Exclusion:** Organization that were not part of this process and have no implicit and explicit knowledge. - **Inclusion:** health workers that participated, enrolled and certificated in the basic, intermediate and advanced FETP program and are surveillance workers. - **Exclusion:** health workers that did not enroll in the FETP program and are not surveillance workers. | Five (5) members representing:  1-person former Minister of Health,  1-person director of health management information system and research in MOH,  1-person director infectious disease epidemiology in NPHIL,  1-persons director general national public health institute and  1-Human Resources MOH |
| II. Government representing non-health sector | A registry of 34 present and past Health Sector Coordinating Committee (HSCC) members | - **Inclusion:** representing people who were part of the one health collaborating platform, representing the animal and education sectors. - **Exclusion:** Organization that were not part of this process and have no implicit and explicit knowledge. | 1-person veterinarian epidemiologist MOA  1-person, surveillance focal person for FETP V-MOH  1-Ministry of Education- MOE |
| III. Donors Agencies | Registry 34 HSCC members who represent the donor agencies/communities. | - **Inclusion:** major donors of the one health collaborating platform that provided resources for the Health system, Ebola response and resiliency. - **Exclusion:** HSCC members that did not play a role at all. | seven (7) members of donor agencies:  1-person current representative WHO-Liberia,  1-person past representative WHO-Liberia,  2-CDC Liberia Country representative past/present,  1-person CDC Anthropologist,  1-person World Bank REDISSE project,  1- USAID health officer |
| IV. Private-for-profit educational sector | 2 private educational institutions | - **Inclusion:** organizations supported strengthening coordination and health information system, from Ebola response to sustainable public Health systems in Liberia | 1-project manager of surveillance project from, Johns Hopkins Bloomberg School of Public Health  1-dean Cuttington University School of Public Health |
| V. Community-Based Organizations (CBOs), | Directory of over (n=120) Liberian Local and International NGOs/CBOs, | - **Inclusion:** intensively participated in the Ebola response, crucial role in IDRS implementation from a directory (n=120) CBOs. - **Exclusion:** CBOs that did not participate in EVD response neither participate in surveillance no intrinsic knowledge. | Five (5) participants representing 5 health regions in 15 counties.  Six (6) Community based organization, |
| VI. Locally registered NGOs (LNGOs) | Directory of over (n=120) Liberian Local and International NGOs/CBOs, | - **Inclusion:** Community-based organization, that intensively participated in the Ebola response, crucial role in IDRS implementation. - **Exclusion:** CBOs that did not participate in EVD response neither participate in surveillance no intrinsic knowledge. | 3 LNGOs/CBOs partners:  1-Executive director, Public Health Initiative of Liberia  1-Resident Coordinator, Africa Field Epidemiology Network  1-CEO of Riders for Health |
| VII. Internationally Registered (INGOs) | Directory of over (n=20) Liberian Local and International INGOs | - INGO, that intensively participated in the Ebola response, crucial role in IDRS implementation. - **Exclusion:** INGOs that did not participate in EVD response neither participate in surveillance no intrinsic knowledge. | 3 INGOs  1-Health Officer International Rescue Committee  1-Country Manager, LastMile Health  1-Health Officer Africa-Liberia |

##### Appendix 4 shows relevant documents identified from MOH, NPHIL and agencies

| Documents selected |
| --- |
| National Health policy and Plan (2011-2021) |
| Investment Plan for Building a Resilient Health System (2015-2021) |
| Essential Package for Health Service |
| Basic Package for Health Service |
| National Action Plan for Health Security (2018) |
| National Health and Social Welfare Financing Policy and Plan (2011-2021) |
| (b) Liberia Health Information System & ICT Strategic Plan (2016-2021) |
| National Public Health Institute Strategic Plan (2017-2022) |
| National M&E Policy and Strategy (2017-2021) |
| The Acts Establishing the National Public Health Institute of Liberia (2016) |
| Liberia Health Information System & ICT Strategic Plan (2016-2021) |
| National Research for Health Policy and Strategy (2018-2023) |
| National Human Resources Policy |
| National Integrated Disease Surveillance guidelines |
| National One Health Policy and Framework |
| National Policy for Antimicrobial Resistance |
| Liberia Joint External Evaluation (JEE) 2016 |
| Field Epidemiologic Training Module |
| Semester Bulletin for Surveillance Activities |
| Ministry of Health Annual Reports (2013-2020) |
| Demographic Health Survey Reports (2019/2020) |
| International Health Regulation Joint External Evaluation for Liberia (2016) |
| Integrated Disease Surveillance & Response Technical Guidelines 3^rd^ edition (2021) |
| One Health Coordination Platform (2017) |
| National Infection Prevention and Control Guidelines (2018) |
| Liberia National Health Quality Strategy (2017 – 2021) |
| National Laboratory 5-year Strategy Plan 2018 |
| Ministry of Agriculture, Animal Disease Surveillance and Response ADSR, First Newsletter, No.2 December 2019 |
| Ministry of Agriculture, First National Animal Disease Surveillance and Response System (ADSR), January 2019 |
| Ministry of Agriculture, Community Animal Health Workers Training Manual |
| Final Report: Understanding Why Ebola Deaths Occur at Home in Urban Montserrado County, Liberia Report on the Findings from a Rapid Anthropological Assessment December 22-31, 2014 |
| Liberia Disease Surveillance, Data Quality Report, Results of Data Quality Audit of Immediately Reportable Diseases, Laboratories, and IDSR Resources June 2017 |

1. . Health Aging Network Summary Report conducted by Macro International) accessible at https://www.cdc.gov/aging/pdf/HAN_case_study_report.pdf. [↑](#endnote-ref-1)
